# Fluorescence Quantum Yield Measurements*

**DOI:** 10.6028/jres.080A.038

**Published:** 1976-06-01

**Authors:** J. B. Birks

**Affiliations:** University of Manchester, Manchester, U.K.

**Keywords:** Fluorescence lifetime, fluorescence quantum efficiency, fluorescence quantum yields, fluorescence spectrum, fluorescence standards, molecular fluorescence parameters, observed (technical) fluorescence parameters, polarization, radiative and non-radiative transition probabilities, real fluorescence parameters

## Abstract

Four *molecular fluorescence parameters* describe the behaviour of a fluorescent molecule in very dilute (~ 10^−6^*M*) solution:

- the fluorescence spectrum $F_{M}\left(\bar{v}\right)$;
- the fluorescence polarization *P_M_*;
- the radiative transition probability *k_FM_*; and
- the radiationless transition probability *k_IM_*.
  These parameters and their temperature and solvent dependence are those of primary interest to the photophysicist and photochemist. $F_{M}\left(\bar{v}\right)$ and *P_M_* can be determined directly, but *k_FM_* and *k_IM_* can only be found indirectly from measurements of the secondary parameters,
- the fluorescence lifetime *τ_M_*, and
- the fluorescence quantum efficiency *q_FM_*,

where *k_FM_*=*q_FM_/τ_M_* and *k_IM_*=(1–*q_FM_*) *τ_M_.*

The *real fluorescence parameters* $F\left(\bar{v}\right)$, *τ* and *ϕ_F_* of more concentrated (*c* > 10^−5^ *M*) solutions usually differ from the molecular parameters $F_{M}\left(\bar{v}\right)$, *τ_M_* and *q_FM_* due to concentration (self) quenching, so that *τ* > *τ_M_* and *ϕ_F_* < *q_FM_.* The concentration quenching is due to excimer formation and dissociation (rates *k_DM_c* and *k_MD_*, respectively) and it is often accompanied by the appearance of an excimer fluorescence spectrum $F_{D}\left(\bar{v}\right)$ in addition to $F_{M}\left(\bar{v}\right)$, so that $F\left(\bar{v}\right)$ has two components. The *excimer fluorescence parameters* $F_{D}\left(\bar{v}\right)$, *P_D_*, *k_FD_* and *k_ID_* together with *k_DM_* and *k_MD_*, and their solvent and temperature dependence, are also of primary scientific interest.

The *observed* (technical) *fluorescence parameters* $F^{T}\left(\bar{v}\right)$, *τ^T^* and $\phi_{F}^{T}$ in more concentrated solutions usually differ from the real parameters $F\left(\bar{v}\right)$, *τ* and *ϕ_F_*, due to the effects of self-absorption and secondary fluorescence. The technical parameters also depend on the optical geometry and the excitation wavelength. The problems of determining the real parameters from the observed, and the molecular parameters from the real, will be discussed.

Methods are available for the accurate determination of $F^{T}\left(\bar{v}\right)$ and *τ^T^*. The usual method of determining $\phi_{F}^{T}$ involves comparison with a reference solution *R*, although a few calorimetric and other absolute determinations have been made. For two solutions excited under identical conditions and observed at normal incidence

$$\frac{\phi_{F}^{T}}{\phi_{FR}^{T}}=\frac{n^{2}\int F^{T}\left(\bar{v}\right)d\bar{v}}{n_{R}^{2}\int F_{R}^{T}\left(\bar{v}\right)d\bar{v}}$$

where *n* is the solvent refractive index.

Two reference solution standards have been proposed, quinine sulphate in *N* H_2_SO_4_ which has no self-absorption, and 9,10-diphenylanthracene in cyclohexane which has no self-quenching. The relative merits of these solutions will be discussed, and possible candidates for an “ideal” fluorescence standard with no self-absorption and no self-quenching will be considered.

## 1. Introduction

Most atoms, molecules, polymers and crystals emit ultraviolet, visible or infrared photons following excitation of their electronic energy levels. This emission or *luminescence* is classified according to the mode of excitation:

*photoluminescence* due to optical (non-ionizing) radiation;
*cathodoluminescence* due to cathode rays (electron beams);
*radioluminescence* (*scintillations*) due to ionizing radiation;
*electroluminescence* due to electric fields;
*thermoluminescence* produced thermally after prior irradiation by other means;
*triboluminescence* due to frictional and electrostatic forces;
*sonoluminescence* due to ultrasonic radiation;
*chemiluminescence* due to a chemical process, commonly oxidation;
*electrochemiluminescence* due to a chemical process, initiated by an electric field; and
*bioluminescence* due to a biological process, usually enzymatic in origin.
*Luminescent materials* can be divided into several broad groups.

- *Aromatic molecules* constitute the largest group. They emit luminescence in the vapour, liquid, polymer and crystal phases and in fluid and rigid solutions [1]^1^. They are used extensively in organic liquid, plastic and crystal scintillators [2], luminescent dyes and paints, detergent and paper whiteners, luminescent screens, dye lasers, etc.
- Many *inorganic crystals*, including diamond, ruby, alkali halides, zinc sulphide and calcium tungstate, luminesce efficiently. The emission is usually from impurity centres (activators) or, in the absence of such impurities, from crystal defects [2]. Luminescent inorganic crystals are used as scintillators [2], luminescent screens, solid-state lasers, jewels, etc.
- *Noble gases* (He, Ne, Ar, Kr, Xe) luminesce in the vapour, liquid, and solid phases and in liquid and solid solutions [2, 3]. They are used in discharge lamps, gas lasers and scintillators.
- Many *simple inorganic molecules* luminesce in the vapour phase [4]. Some, like H_2_, D_2_, N_2_, and Hg are used in discharge lamps; others, like N_2_, I_2_, and CO_2_ are used in gas lasers.
- Some *inorganic ions*, notably those of the rare earth elements, are luminescent. They are used as activators in inorganic crystals (see (ii) above), glasses and chelates. Applications include inorganic crystal and glass scintillators and Nd glass lasers.
- Many *biological molecules* are luminescent. These include
  - *aromatic amino-acids* (tryptophan, tyrosine, phenylalanine) in proteins;
  - *nucleotides* (adenine, guanine, uracil, cytosine, thymine) in DNA and RNA;
  - *retinyl polyenes* in the visual pigments;
  - *chlorophylls* and *carotenoids* in the photosynthetic chloroplast; and
  - several *vitamins* and *hormones.*
  The study of biomolecular luminescence is an important area of biophysical research [5].
- *Aliphatic molecules*, such as the paraffins and cyclohexane, once considered to be nonluminescent, are now known to emit in the far ultraviolet (~ 200 nm) with low quantum yield [6]. This list, which is not exhaustive, illustrates the wide range of luminescent materials and their applications.

## 2. Luminescence of Aromatic Molecules

### 2.1. Radiative transitions

The initial discussion is limited to aromatic molecules (i), but it will be later extended to other luminescent materials (ii)–(vii). Most aromatic molecules have an even number of *π*-electrons, giving a ground singlet electronic state *S*_0_ in which the electron spins are paired. The *excited π* electronic states of the molecule are either

singlet states: *S*_1_, *S*_2_ … *S_p_;* or
triplet states: *T*_1_, *T*_2_ … *T_q_.*

A spin-allowed radiative transition (luminescence) between two states of the same multiplicity (e.g. *S*_1_ → So, *S_p_* → *S*_0_, *T_q_* → *T*_1_) is called *fluorescence* (*F*). A spin-forbidden radiative transition between two states of different multiplicity (e.g. *T*_1_→ *S*_0_) is called *phosphorescence* (P). The energy difference between the initial and final electronic state is emitted as a fluorescence photon (*hv_F_*) or phosphorescence photon (*hv_p_*).

The fluorescence occurring immediately after the initial excitation of *S*_1_ (or *S_p_*) is known as *prompt fluorescence.* In some molecules or molecular systems there are mechanisms by which *S*_1_ (or *S_p_*) may become excited subsequent to the initial excitation, resulting in *delayed fluorescence.* The two principal mechanisms are as follows [1].

- Thermal activation of molecules in the lowest triplet state *T*1, which is long-lived because the *T*_1_ → *S*_0_ transition is spin-forbidden, repopulates the fluorescent singlet state *S*_1_, resulting in *E-type* (eosin-type) *delayed fluorescence*, so called because it occurs in eosin and other dye molecules.
- Diffusional interaction between pairs of *T*_1_-excited molecules in solution or *T*_1_ excitons in a crystal creates singlet-excited molecules by the process
  $$T_{1}+T_{1}\to S_{1}\;\left(\text{or}\;S_{p}\right)+S_{0}$$ (1)
  resulting in *P-type* (pyrene-type) *delayed fluorescence*, so called because it occurs in pyrene and other aromatic hydrocarbons.

### 2.2. Radiationless Transitions

Radiative transitions are between electronic states of different energy. In a complex molecule or crystal there are also radiationless transitions between different electronic states of the same energy. These isoenergetic radiationless transitions are induced by molecular or crystal vibrations.

A spin-allowed radiationless transition between two states of the same multiplicity is called *internal conversion* (*IC*). A spin-forbidden radiationless transition between two states of different multiplicity is called *intersystem crossing* (*ISC*).

### 2.3. Vibrational Relaxation

After the initial excitation or after an isoenergetic radiationless transition, the molecule is usually in a vibronic state $S_{p}^{*}$ (or $T_{q}^{*}$) corresponding to a vibrationally-excited level of a particular electronic state *S_p_* (or *T_q_*). In a condensed medium (solution, liquid, polymer, crystal) or a high-pressure vapour the excess vibrational energy $S_{p}^{*}-S_{p}^{o}$ (or $T_{q}^{*}-T_{q}^{o}$) is rapidly dissipated collisionally to the environment leading to *vibrational relaxation* (*VR*).

The dissipative *VR* process, which is distinct from the nondissipative *IC* and *ISC* processes, plays an essential role in the thermal equilibration of the excited molecules. At normal temperatures *VR* is rapid (~ 10^−12^–10^−13^ s, depending on the excess vibrational energy to be dissipated) and much faster than *IC*, *ISC*, *F* or *P.*

Isolated excited molecules in a low-pressure vapour, where *VR* is inhibited by the low collision rate, behave in a different manner than those in the condensed phase [6]. In an isolated molecule the fluorescence occurs from the vibronic state $S_{p}^{*}$ initially excited or from isoenergetic vibronic states $S_{1}^{*}$, $S_{2}^{*}\cdots .$ of lower electronic states populated by *IC.* This phenomena is called *resonance fluorescence.* In the condensed phase *VR* brings the excited molecules rapidly into thermal equilibrium and all the processes (*F*, *P*, *IC* and *ISC*) occur from an equilibrated system of molecules.

### 2.4. Photophysical Processes and Parameters

**Figure 1:**
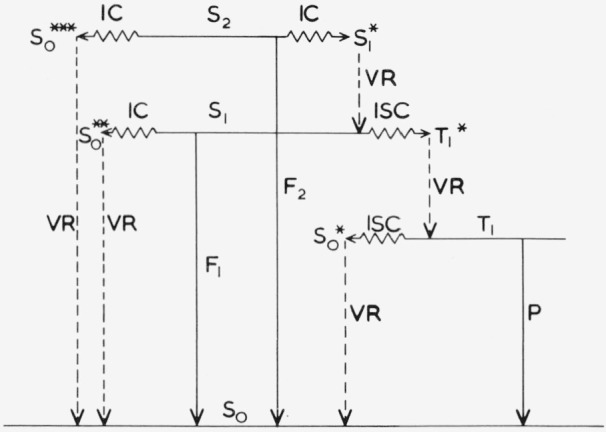
Schematic diagram of radiative (solid vertical lines), radiationless (wavy horizontal lines), and vibrational relaxation (broken vertical lines) transitions between electronic states (solid horizontal lines) *S_2_, S_1_, T_1_* and *S_0_* of an aromatic molecule in a condensed medium. *F* = fluorescence, *P* = phosphorescence, *IC* = internal conversion, *ISC* = intersystem crossing, *VR* = vibrational relaxation.

Figure 1 shows schematically the photophysical processes that can occur in an aromatic molecular system in very dilute solution (~ 10^−6^ *M*) following excitation into *S*_2_.

*S*_2_ decays by

- *IC* to $S_{1}^{*}$, followed by *VR* to *S*_1_;
- *IC* to $S_{0}^{***}$, followed by *VR* to *S*_0_; or
- *S*_2_ → *S*_0_ fluorescence *F*_2_.
  *S*_1_*→S*_1_ fluorescence, which could potentially occur, is forbidden since *S*_2_ and S_1_ have the same parity (*ungerade*) [1].
  *S*_1_, from (a), decays by
- *S*_1_ → *S*_0_ fluorescence *F*_1_;
- *ISC* to $T_{1}^{*}$, followed by *VR* to *T*_1_; or
- *IC* to $S_{0}^{**}$, followed by *VR* to *S*_0_.
  *T*_1_, from (e), decays by
- *T*_1_ → *S*_0_ phosphorescence *P*; or
- *ISC* to $S_{0}^{*}$, followed by *VR* to *S*_0_

**Figure 2:**
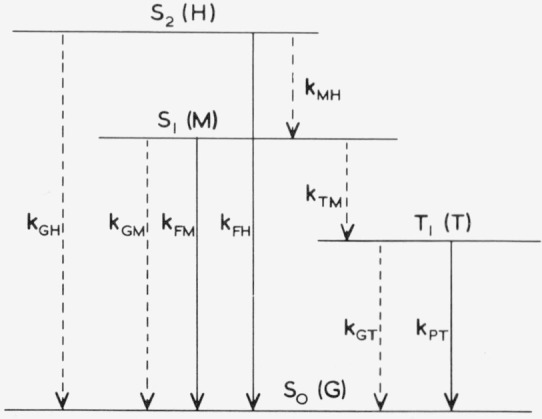
Rate parameters of radiative transitions (solid vertical lines) and radiationless plus vibrational relaxation transitions (1broken vertical lines) between electronic states (solid horizontal lines) *S_2_, S_1_, T_1_*, and *S_0_* of an aromatic molecule in a condensed medium. The notation of the states, radiations and rate parameters is indicated.

*F*, *P*, *IC* and *ISC* are the rate-determining processes, since *VR* is much faster, *k_AB_* is defined as the rate parameter of the *B → A* process, where *B* is the initial state and *A* is the product radiation (*F* or *P*) or final state (for *IC* or *ISC*) [1]. Subscripts *G* = *S*_0_, *T*=*T*_1_, *M* = *S*_1_, and *H* = *S*_2_ indicate the different states. Figure 2 shows the rate parameters corresponding to the processes of figure 1. In the rate parameter description the *VR* subsequent to each *IC* or *ISC* is omitted, but the distinction between the isoenergetic radiationless transitions and the vibrational relaxation should not be overlooked.

The *S*_2_, *S*_1_ and *T*_1_ *decay parameters* are given by

$$k_{H}=k_{FH}+k_{MH}+k_{GH}=1/\tau_{H}$$ (2)

$$k_{M}=k_{FM}+k_{TM}+k_{GM}=1/\tau_{M}$$ (3)

$$k_{T}=k_{PT}+k_{GT}=1/\tau_{T}$$ (4)

where *τ_H_*, *τ_M_* and *τ_T_* are the *S*_2_, *S*_1_ and *T*_1_ *lifetimes*, respectively.

The *quantum efficiency q_AB_* of any photophysical process, rate *k_AB_*, from an excited state *B* is defined as the fraction of the excited molecules in *B* that decay by that process, so that

$$q_{AB}=k_{AB}/k_{B}$$ (5)

The *S*_2_ → *S*_0_ and *S*_1_ → *S*_0_ *fluorescence quantum efficiencies* are, respectively.

$$q_{FH}=k_{FH}/k_{H}$$ (6)

$$q_{FM}=k_{FM}/k_{M}$$ (7)

the *T*_1_ → S_0_ phosphorescence quantum efficiency is

$$q_{PT}=k_{PT}/k_{T}$$ (8)

the $S_{2}\to S_{1}^{*}$ internal conversion quantum efficiency is

$$q_{MH}=k_{MH}/k_{H}$$ (9)

and the $S_{1}\to T_{1}^{*}$ and $T_{1}\to S_{1}^{*}$ *intersystem crossing quantum efficiencies* are, respectively,

$$q_{TM}=k_{TM}/k_{M}$$ (10)

$$q_{GT}=k_{GT}/k_{T}$$ (11)

The rate parameters (fig. 2), the decay parameters and lifetimes (2)–(4), and the quantum efficiencies (5)–(11) are *molecular parameters.* They refer to very dilute (~ 10^−6^M) solutions, containing no dissolved oxygen or other impurity quenchers.

An increase in the solution molar concentration *c* does not change the unimolecular rate parameters, but it introduces bimolecular processes due to interactions between excited molecules in *S*_2_, *S*_1_ or *T*_1_ and unexcited molecules in So, producing *concentration quenching.* To a first approximation the *S*_2_, *S*_1_ and *T*_1_ concentration quenching rates may be expressed as *k_CH_c*, *k_CM_c* and *k_CT_c*, and the *S*_2_, *S*_1_ and *T*_1_ decay parameters become

$$k_{H}^{\prime }=k_{H}+k_{CH}c=1/\tau_{H}^{\prime }$$ (2a)

$$k_{M}^{\prime }=k_{M}+k_{CM}c=1/\tau_{M}^{\prime }$$ (3a)

$$k_{T}^{\prime }=k_{T}+k_{CT}c=1/\tau_{T}^{\prime }$$ (4a)

respectively, where $\tau_{H}^{\prime }$, $\tau_{M}^{\prime }$ and $\tau_{T}^{\prime }$ are the *S*_2_, *S*_1_ and *T*_1_ lifetimes in a solution of molar concentration *c*. An exact treatment also considers the rate parameters of the *excimers* produced by the concentration quenching and their dissociation [1], but the Stern-Volmer approximation of (2a)–(4a) is adequate for the present discussion.

The *quantum yield ϕ* of any photophysical process in a solution of concentration *c* is defined in the same manner as the quantum efficiency, except that the limitation to very dilute solutions is removed. The *S*_2_ → *S*_0_ and *S*_1_ → *S*_0_ *fluorescence quantum yields* are, respectively

$$\phi_{FH}=\frac{k_{FH}}{k_{H}+k_{CH}c}=\frac{q_{FH}}{1+K_{CH}c}$$ (12)

$$\phi_{FM}=\frac{k_{FM}}{k_{M}+k_{CM}c}=\frac{q_{FM}}{1+K_{CM}c}$$ (13)

and the *T*_1_ → *S_0_ phosphorescence quantum yield* is

$$\phi_{PT}=\frac{k_{PT}}{k_{T}+k_{CT}c}=\frac{q_{PT}}{1+K_{CT}c}$$ (14)

The parameters *K_CH_*(= *k_CH_*/*k_H_*), *K_CM_*(= *k_CM_*/*k_M_*) and *K_CT_*(*k_CT_*/*k_T_*) are the *Stern-Volmer coefficients of concentration quenching of S*_2_, *S*_1_ and *T*_1_, respectively.

The $S_{2}\to S_{1}^{*}$ *internal conversion quantum yield* is

$$\phi_{MH}=\frac{k_{MH}}{k_{H}+k_{CH}c}=\frac{q_{MH}}{1+K_{CH}c}$$ (15)

and the $S_{1}\to T_{1}^{*}$ and $T_{1}\to S_{0}^{*}$ *intersystem crossing quantum yields* are, respectively,

$$\phi_{TM}=\frac{k_{TM}}{k_{M}+k_{CM}c}=\frac{q_{TM}}{1+K_{CM}c}$$ (16)

$$\phi_{GT}=\frac{k_{GT}}{k_{T}+k_{CT}c}=\frac{q_{GT}}{1+K_{CT}c}$$ (17)

The above expressions for quantum efficiencies and yields all refer to direct excitation of the state from which the process occurs, and they require revision when the state is not excited directly. Thus for excitation into *S*_2_, the *S*_1_ → *S*_0_ fluorescence quantum yield is

$$\phi_{FM}^{H}=\phi_{MH}\phi_{FM}$$ (18)

For excitation into *S*_1_, the *T*_1_ → *S*_0_ phosphorescence quantum yield is

$$\phi_{PT}^{M}=\phi_{TM}\phi_{PT}$$ (19)

### 2.5. Vavilov’s Law and Kasha’s Rules

It is commonly assumed that *ϕ_MH_* = 1.0 for $S_{2}\to S_{1}^{*}$ *IC* and that *ϕ*=1 for *IC* between higher excited states within the singlet (*S_p_*) manifold, so that *ϕ_FM_* is independent of the excitation wavelength *λ*_ex_ up to the ionization potential. This assumption, known as *Vavilov’s law*, has been confirmed for many compounds in solution. Major deviations from Vavilov’s law have, however, been observed for solutions of benzene, toluene, p-xylene, mesitylene, fluorobenzene, naphthalene, 2-methylnaphthalene, 1,6-dimethylnaphthalene [1], tryptophan, tyrosine and phenylalanine [7]. In each case it is observed that $\phi_{FM}^{H}/\phi_{FM}=\phi_{MH}<1$. In benzene and its derivatives and possibly in the other compounds, the effect is due to efficient $S_{2}\to S_{0}^{***}$ *IC* (*k_GH_*) competing with $S_{2}\to S_{1}^{*}$ *IC* (*k_MH_*) [8]. In fluorescence quantum yield measurements it is essential either to verify that Vavilov’s law applies, or to limit the excitation to the region of the *S*_0_ → *S*_1_ absorption spectrum.

*Kasha’s rules* [9], another well-known generalization, state that in a complex molecule luminescence occurs only from the lowest excited state of a given multiplicity, i.e., *S*_1_ → *S*_0_ fluorescence and *T*_1_ → *S*_0_ phosphorescence. For many years azulene and its derivatives, which emit *S*_2_ → *S*_0_ fluorescence and negligible *S*_1_ → *S*_0_ fluorescence, were the main exceptions to Kasha’s rules. Recently the picture has changed dramatically.

In addition to the normal *S*_1_ → *S*_0_ fluorescence, weak *S*_2_ → *S*_0_ fluorescence has been observed in benzene, toluene, *p*-xylene, mesitylene, naphthalene, pyrene, 1:2-benzanthracene, 3:4-benzopyrene, 1:12-benzoperylene and ovalene, weak *S*_3_ → *S*_0_ fluorescence has been observed in *p*-xylene, mesitylene, naphthalene, pyrene and 1:2-benzanthracene, and weak *S*_4_ → *S*_0_ fluorescence has been observed in pyrene and fluoranthene [6, 10].

Such fluorescence from higher excited states was predicted by the author in 1954 [11]. Its detection is difficult, since it occurs in the region of the *S*_0_ → *S_p_* absorption spectrum, and its quantum yield is only ~ 10^−5^ *ϕ_FM_* [6]. Subsequent attention will be focused on the main *S*_1_ → *S*_0_ fluorescence.

### 2.6. The Fluorescence Spectrum

The *S*_1_ → *S*_0_ fluorescence spectrum occurs from a system of *S*_1_ excited molecules in thermal equilibrium in solution. The fraction of these molecules with vibrational energy *E_v_* is proportional to exp (−*E_v_/kT*), where *k* is Boltzmann’s constant and *T* is the absolute temperature. A large majority are in the zero point level $S_{1}^{0}$, and to a first approximation the fluorescence of the “hot” molecules can be disregarded.

The $S_{1}^{0}\to S_{0}$ fluorescence occurs into $S_{0}^{0}$, the zeropoint level of *S*_0_, and into the many vibrational levels of So. The $S_{1}^{0}\to S_{0}^{0}$ transition, or *0–0 fluorescence transition*, of wavenumber ${\left({\bar{v}}_{00}\right)}_{F}$ is the highest energy transition in the $S_{1}^{0}\to S_{0}$ fluorescence spectrum. In the vapour ${\left({\bar{v}}_{00}\right)}_{F}$ coincides with ${\left({\bar{v}}_{00}\right)}_{A}$, the corresponding $S_{0}^{0}\to S_{1}^{0}$ *0–0 absorption transition.* In solution, due to solvent polarization effects

$${\left({\bar{v}}_{00}\right)}_{A}-{\left({\bar{v}}_{00}\right)}_{F}=\Delta {\bar{v}}_{00}$$ (20)

where $\Delta {\bar{v}}_{00}$ varies from 0 to a few hundred cm^−1^ depending on the solvent [1]. In benzene the 0–0 fluorescence and absorption transitions are symmetry-forbidden and they are absent from the vapour spectra. They appear as weak solvent-induced bands (the Ham bands) in solution spectra, the intensity depending on the solvent [1].

At low temperatures the *S*_1_ → *S*_0_ $\left(=S_{1}^{0}\to S_{0}\right)$ *fluorescence spectrum* $F_{M}\left(\bar{v}\right)$ consists of a complex series of a few hundred narrow lines of different intensities, which may be analysed into progressions and combinations of the different vibrational modes of the unexcited molecule. When the temperature is increased, thermal broadening and solvent-solute interactions obscure most of the vibrational structure. At room temperature $F_{M}\left(\bar{v}\right)$ commonly consists of a few prominent broad bands with little other structure. Thus $F_{M}\left(\bar{v}\right)$ for anthracene in cyclohexane solution consists of a progression of 5 broad bands, spaced about 1400 cm^−1^ apart, corresponding to *CC* vibrational modes. Similar vibrational progressions occur in $F_{M}\left(\bar{v}\right)$ for other condensed hydrocarbons [1], For large molecules, e.g., dyes, with many degrees of vibrational and/or rotational freedom, $F_{M}\left(\bar{v}\right)$ at room temperature often consists of a single broad band with no vibrational structure. Berlman [12] has recorded the fluorescence spectra of many aromatic molecules.

The solvent has a strong influence on $F_{M}\left(\bar{v}\right)$ at room temperature. In a polar solvent like ethanol the vibrational bands are broad and poorly resolved, and the separation $\Delta {\bar{v}}_{00}$ between the absorption and fluorescence 0–0 hands is relatively large. In a nonpolar aliphatic hydrocarbon solvent, like cyclohexane or *n*-hexane, the spectral resolution is improved and $\Delta {\bar{v}}_{00}$ is reduced. In a fluorocarbon solvent, like perfluoro-*n*-hexane (PFH), each of the vibrational bands has a well-resolved fine structure, similar to that in the vapour phase, and $\Delta {\bar{v}}_{00}=0$ [13]. PFH is an ideal spectroscopic solvent, apart from cost and the low solubility of aromatic molecules in PFH.

At temperatures above about −100 °C the “hot” vibrationally-excited *S*_1_ molecules with a Boltzmann distribution of energies $S_{1}^{*}\left(=S_{1}^{0}+E_{v}\right)$ also contribute to $F_{M}\left(\bar{v}\right)$. Each component $S_{1}^{*}\to S_{0}$ spectrum is similar to the $S_{1}^{0}\to S_{0}$ spectrum, except that it is shifted by an amount *E_v_* towards higher energies, and its intensity is proportional to exp (−*E_v_/kT*). Most of the $S_{1}^{*}\to S_{0}$ spectral distribution lies below the $S_{1}^{0}\to S_{0}$ spectrum and is obscured thereby. However, each component $S_{1}^{*}\to S_{0}$ spectrum extends beyond $\Delta {\bar{v}}_{00}$ to $\Delta {\bar{v}}_{00}+E_{v}$, giving rise to *hot fluorescence bands*, the intensity and extent of which increase with temperature. These hot fluorescence bands, which are an integral part of the *S*_1_ → *S*_0_ fluorescence spectrum $F_{M}\left(\bar{v}\right)$ at room temperature, occur in all aromatic molecules, although they are not often recorded. The emission bands are in the same region as the *S*_0_ → *S*_1_ absorption, and special care is needed to observe them [6].

### 2.7. The rate parameters

Observations of *q_FM_* and *τ_M_* for a very dilute solution enable

$$k_{FM}=q_{FM}/\tau_{M}$$ (21)

$$k_{IM}=k_{FM}+k_{GM}=\left(1-q_{FM}\right)\tau_{M}$$ (22)

to be determined. Birks and Munro [14] have reviewed methods of measuring *τ_M_.* Observations of *q_TM_* (= *k_TM_*/*k_M_*), by one of the several methods described by Wilkinson [15], enable *k_TM_* and *k_GM_* to be evaluated. The measurement of *q_PT_* and *τ_T_* permits *k_PT_* and *k_GT_* to be determined [1]. Thus measurements of five quantities *q_FM_*, *τ_M_*, *q_TM_*, *q_PT_* and *τ_T_* are required to determine the five *S*_1_ and *T*_1_ unimolecular rate parameters *k_FM_*, *k_TM_*, *k_GM_*, *k_PT_* and *k_GT_.*

Observations of $\tau_{M}^{\prime }$ and $\tau_{T}^{\prime }$ (or *ϕ_FM_* and *ϕ_PT_*) as a function of the molar concentration *c* enable the bimolecular rate parameters *k_CM_* and *k_CT_* to be determined. The observations and analysis may be extended further to obtain the fluorescence (*k_FD_*), *ISC* (*k_TD_*), *IC*(*k_GD_*) and dissociation (*k_MD_*) rate parameters of the singlet excimer [1]. This involves observations of the molecular (*ϕ_FM_*) and excimer (*ϕ_FD_*) fluorescence quantum yields of concentrated solutions.

It is the *rate parameters* and their dependence on temperature, solvent, substitution etc. that are the quantities of interest to the photophysicist and photochemist, and not the properties from which they are derived. The latter may be of technical interest for particular applications. Of the three quantities *q_FM_*, *τ_M_* and *q_TM_* required to determine the *S*_1_ rate parameters *k_FM_*, *k_TM_* and *k_GM_* the published values of q*_FM_* (or *ϕ_FM_* which is often implicitly equated to *q_EM_*) show the largest scatter. When the solution concentration *c* is increased, self-absorption effects introduce difficulties in the determination of *ϕ_FM_.* It is hoped that this paper will help to improve the situation.

### 2.8. The Fluorescence Rate Parameter

A theoretical expression for *k_FM_* has been derived from the Einstein radiation relation using the zero-order Born-Oppenheimer approximation [16, 17]

$$k_{FM}^{t}=2.88\times 10^{-9}\frac{n_{F}^{3}}{n_{A}}{\langle {\bar{v}}_{F}^{-3}\rangle }_{\text{Av}}^{-1}\int \frac{\epsilon \left(\bar{v}\right)d\bar{v}}{\bar{v}}$$ (23)

where *n_F_* and *n_A_* are the mean refractive indices of the solvent over the *S*_1_→*S*_0_ fluorescence and *S*_0_→*S*_1_ absorption spectra, respectively, ${\langle {\bar{v}}_{F}^{-3}\rangle }_{\text{Av}}^{-1}$ is the reciprocal of the average value of ${\bar{v}}^{-3}$ over the fluorescence spectrum, $\epsilon \left(\bar{v}\right)$ is the decadic molar extinction coefficient, and the integral is taken over the *S*_0_→*S*_1_ absorption spectrum. Relation (23) has been tested for a number of molecules, and excellent agreement between *k_FM_* and $k_{FM}^{t}$ has been obtained for several molecules in different laboratories [1, 12, 16, 17, 18]. Such molecules may be useful as fluorescence standards.

If the solvent optical dispersion is small *n_F_* ≃ *n_A_* = *n*, and (23) can be simplified to

$$k_{FM}^{t}=n^{2}{\left(k_{FM}^{t}\right)}_{0}$$ (24)

where $\left(k_{FM}^{t}\right)$ is a molecular constant, independent of the solvent and the temperature. Relation (24) has been verified for several solutes in different solvents over a wide temperature range [19].

In some molecules there are large discrepancies between *k_FM_* and $k_{FM}^{t}$. A detailed study of these anomalies has revealed the presence of electronic states not observed spectroscopically [20, 21]. The nature and origin of such radiative lifetime anomalies are discussed elsewhere [22]. The factors determining the other *S*_1_ and *T*_1_ rate parameters *k_TM_*, *k_GM_*, *k_PT_* and *k_GT_* have been considered previously [1, 6, 8].

### 2.9. Molecular Fluorescence Parameters

The *S*_1_ → *S*_0_ fluorescence of an aromatic compound in very dilute solution is characterized by the following molecular parameters.

- The fluorescence spectrum $F_{M}\left(\bar{v}\right)$ depends on the solvent and temperature (see 2.6).
- The fluorescence polarization *p_M_* depends on the direction of the transition dipole moment relative to the molecular axes. For a π^*^ → *π* electronic transition this lies in the molecular plane along one of two orthogonal axes depending on the symmetry of *S*_1_. For naphthalene the fluorescence is long-axis polarized; for anthracene it is short-axis polarized [1].
- The fluorescence rate parameter *k_FM_* is proportional to the square of the transition dipole moment [1]. In the absence of any anomalies *k_FM_/n^2^* is independent of the solvent and temperature (24).
- The *S*_1_ radiationless rate parameter *k_IM_* (= *k_TM_ + k_GM_*) describes the processes competing with the fluorescence. *k_IM_* usually depends markedly on the solvent and on the temperature [1].
$F_{M}\left(\bar{v}\right)$ and *p_M_* can be observed directly. The evaluation of *k_FM_* and *k_IM_* involves measurements of two secondary parameters:
- The fluorescence lifetime *τ*_M_; and
- the fluorescence quantum efficiency *q_FM_.*
  Several accurate methods are available for measuring *τ_M_* [14]. Reliable methods are available for measuring *q_FM_*, but they are often used incorrectly [23].
  The molecular fluorescence parameters $F_{M}\left(\bar{v}\right)$, *p*_M_, *k_FM_* and *k_IM_* are independent of the molar concentration *c.* The secondary fluorescence parameters *τ*_M_ and *ϕ_FM_* decrease with increase in *c* due to
- the concentration quenching rate parameter *k_CM_.*

*k_CM_*, which depends markedly on the solvent viscosity and the temperature, is a further molecular parameter of photophysical interest.

## 3. Other Luminescent Materials

The preceding discussion of the luminescence of aromatic molecules is applicable to the other luminescent materials considered in the Introduction. It applies directly to biological molecules (vi) and aliphatic organic molecules (vii). Noble gases (iii) also have singlet ground states, and there are close analogies between them and the aromatic hydrocarbons, particularly in excimer formation [3]. There are no radiationless transitions in the noble gases (*q_FM_*= *q_FH_*= 1.0) because of the absence of internal vibrations. They form excimers in the vapour, liquid, and solid phases, and the vibrational modes of these may generate radiationless transitions and vibrational relaxation in the condensed phase [3].

Simple inorganic molecules (iv) are similar. They normally have singlet ground states and excited singlet and triplet states. Although they have internal vibrations, the vibronic state density is low, and there are normally no radiationless transitions except at high excitation energies, where predissociation may occur [4].

The luminescence of inorganic crystals (ii) and inorganic ions (v) in a solid matrix is closely related to that of aromatic molecular crystals. Unfortunately there are major terminological differences between inorganic crystal photophysics and organic molecular crystal photophysics. Table 1 is based on a brief survey of the inorganic luminescence literature, and may require revision in the light of any recent changes.

The inorganic luminescence terminology predates the discovery of electron spin, and it has not been adjusted to take account of this. Because of spin, processes 1(a) and 1(b) differ in lifetime by a factor of up to 10^8^, and it would seem appropriate to distinguish them. In 1933 Jablonski [24], the originator of figure 1, showed that the two slow emissions 1(b) and 1(c) observed in organic dyes originated from a common metastable state *X*, and he proposed that they be called *β*-phosphorescence and *α*-phosphorescence, respectively. Since 1944 when Lewis and Kasha [25] demonstrated that *X*=*T*_1_, the lowest excited triplet state, 1(b) has been called simply phosphorescence, while 1(c) which has the same emission spectrum as 1(a) is called E-type delayed fluorescence.

Standardization of luminescence terminology is long overdue. Those responsible for organizing international luminescence conferences and publishing luminescence journals have unfortunately neglected to formulate a scientific language common to workers in organic and inorganic luminesqence. Perhaps the National Bureau of Standards can assist in the matter.

## 4. Fluorescence Measurements

### 4.1. Fluorescence Spectra

A true (corrected) fluorescence spectrum is plotted as the relative quantum intensity $F_{M}\left(\bar{v}\right)$ (relative number of quanta per unit wave-number interval) against wavenumber $\bar{v}$. A few spectrometers have been developed which record directly the true fluorescence spectrum. The majority provide spectra which require correction for the dispersion of the analyzing monochromator, the spectral response of the photomultiplier or detector, and any light losses. This involves the preparation of an *instrumental calibration curve*, by measurements

- with a calibrated lamp through a neutral filter;
- with a thermopile or bolometer;
- of reference solution fluorescence spectra [26]; or
- with a fluorescent quantum counter.

A *quantum counter* is a system which has a constant fluorescence quantum yield over a broad spectral range. To achieve this it should have a high and relatively constant absorption over the spectral range of interest, it should have negligible self-absorption (no overlap of fluorescence and absorption spectrum), it should obey Vavilov’s law, and it should be stable photochemically. Systems commonly used as quantum counters include:

- 3 gl^−1^ Rhodamine B in ethylene glycol (210–530 nm),
- 4 gT^−1^ quinine sulphate in *N* H_2_SO_4_ (220–340 nm), and
- 10^−2^*M* 1-dimethylaminonaphthalene 5-(or 7-) sodium sulphonate in 0.1 *N* Na_2_CO_3_ (210–400 nm).

An extension of this list would be advantageous.

Three common optical geometries are used in fluorescence measurements;

- front-surface or *reflection geometry*, in which the fluorescence from the irradiated surface of the specimen is observed;
- *90° geometry*, in which the fluorescence is observed in a direction normal to the incident beam; and
- *transmission geometry*, in which the fluorescence is observed from the opposite side of the speciment to the excitation.

For very dilute solutions (~ 10^−6^M) the three geometries give the same fluorescence spectrum, quantum efficiency and lifetime. The 90° geometry, used by Birks and Dyson [17] and others, has the advantage of minimizing background incident light and of allowing the fraction of incident light absorbed in the specimen to be monitored directly.

An increase in the solution concentration *c* reduces *q_FM_* and *τ_M_* to *ϕ_FM_* and $\tau_{M}^{\prime }$, respectively, due to concentration quenching. It also attenuates the high-energy region of $F_{M}\left(\bar{v}\right)$ due to self-absorption arising from the overlap of the absorption and fluorescence spectra. As *c* is increased the intensity of the 0–0 fluorescence band decreases towards zero due to its overlap with the 0–0 absorption band. At room temperature and high *c* the self-absorption may extend to the 0–1 and 0–2 fluorescence bands, which overlap the 1–0 and 2–0 hot absorption bands, due to thermally activated molecules in the first and second vibrational levels of *S*_0_. These self-absorption effects are a maximum in the transmission geometry (c), somewhat reduced in the 90° geometry (b), and they are least in the reflection geometry (a), which is normally used for fluorescence studies of more concentrated solutions.

The effect of self-absorption on $F_{M}\left(\bar{v}\right)$ observed in reflection can be minimized by Berlman’s technique [12] of excitation at an intense absorption maximum, thereby minimizing the penetration depth *d*_ex_ of the exciting light. This technique does not, however, compensate for the secondary fluorescence produced by the self-absorption and which modifies *ϕ_FM_* and *τ_M_*, as discussed below.

### 4.2. Fluorescence Quantum Yields

Absolute determinations of fluorescence quantum yields have been made using integrating spheres to collect the fluorescence emission over a full 4*π* solid angle, by calorimetry to distinguish radiative processes from radiationless processes and vibrational relaxation, by actinometry to integrate fight intensities photochemically, and by polarization and scattering measurements. These methods have been reviewed by Lipsett [27] and Demas and Crosby [28].

The superscript *T* is introduced to refer to the *observed* (*technical*) *fluorescence parameters* $F_{M}^{T}\left(\bar{v}\right)$, $\phi_{FM}^{T}$ and $\tau_{M}^{T}$, which may differ from the true fluorescence parameters $F_{M}\left(\bar{v}\right)$, *ϕ_FM_* and $\tau_{M}^{\prime }$, due to self-absorption and secondary fluorescence. Absolute determinations of *ϕ_FM_* are difficult and uncommon, and it is normal practice to measure $\phi_{FM}^{T}$ by comparison with a standard of known fluorescence quantum yield $\phi_{FR}^{T}$. If $F_{M}^{T}\left(\bar{v}\right)$ and $F_{T}^{T}\left(\bar{v}\right)$ are the corrected fluorescence spectra of the specimen and standard, respectively, excited under identical conditions (same excitation wavelength, optical density and geometry) and observed *at normal incidence in reflection*, then

$$\frac{\phi_{FM}^{T}}{\phi_{FR}^{T}}=\frac{n^{2}\int_{0}^{\infty }F_{M}^{T}\left(\bar{v}\right)d\bar{v}}{n_{R}^{2}\int_{0}^{\infty }F_{R}^{T}\left(\bar{v}\right)d\bar{v}}$$ (25)

where *n* and *n_R_* are the refractive indices of the specimen solution and the standard solution, respectively. The integrations are often made using a quantum counter [28].

The refractive index term is a correction for the solution optical geometry. The angular dependence of the fluorescence flux *F*(*ϕ*) from a small isotropically emitting source behind an infinite plane surface in a medium of refractive index *n* is

$$F\left(\phi \right)=F_{0}\left(\cos\phi \right)n^{-1}{\left(n^{2}-\sin^{2}\phi \right)}^{-1/2}$$ (26)

where *F*_0_ is a constant $\left(\propto \phi_{FM}^{T}\right)$ and *F*(*ϕ*) is the flux (in quanta cm^2^ s^−1^) falling on a small aperture at an angle *ϕ* from the normal to the face. For *ϕ* = 0° (26) reduces to

$$F\left(0\right)=F_{0}/n^{2}$$ (27)

leading to (25). Relation (26) has been verified by Melhuish [29] who recommended the use of cuvettes with blackened back and sides for fluorescence yield measurements to minimize internal reflection errors.

Shinitzky [30] has pointed out a further potential source of error in fluorescence quantum yield and lifetime measurements. When a fluorescent system is excited by unpolarized fight and its emission is detected without a polarizer, the emission intensity has a typical anisotropic distribution which is directly related to its degree of polarization. This effect can introduce an error of up to 20 percent in all fluorescence quantum yield and lifetime measurements, but it is eliminated when the fluorescence is detected at an angle of 55° or 125° to the direction of excitation, provided that the emission detection system is unbiased with respect to polarization. Procedures for the elimination of polarization errors for partially polarized excitation and biased detection systems were developed by Cehelnik, Mielenz, and Velapoldi [31] and Mielenz, Cehelnik, and McKenzie [32].

If *n* and *n_R_* differ, it is recommended that the specimen and reference solutions be excited at 55° incidence angle and observed at normal incidence, to eliminate the polarization effect and simplify the refractive index correction. The latter correction disappears if *n* = *n_R_*, and the excitation and front-face observation directions need only differ by 55°. The angles of incidence and “reflection” should differ to minimize scattered light.

The self-absorption attenuates the high-energy end of $F_{M}\left(\bar{v}\right)$, but it does not affect the low-energy end. If $F_{M}\left(\bar{v}\right)$, observed in very dilute solution, and $F_{M}^{T}\left(\bar{v}\right)$, observed at molar concentration *c*, are *normalized* in the low-energy region, then the parameter

$$a=\frac{A_{M}-A_{M}^{T}}{A_{M}}$$ (28)

where

$$A_{M}=\int_{0}^{\infty }F_{M}\left(\bar{v}\right)d\bar{v}$$ (29)

$$A_{M}^{T}=\int_{0}^{\infty }F_{M}^{T}\left(\bar{v}\right)d\bar{v}$$ (30)

represents the *self-absorption probability.* This normalization procedure, introduced for anthracene crystal fluorescence [33], has been applied by Birks and Christophorou [34] to concentrated solutions of aromatic hydrocarbons. Substitution of *A_M_* in place of $A_{M}^{T}$ in (25) gives *ϕ_FM_* in place of $\phi_{FM}^{T}$ For materials of low *ϕ_FM_* (< 0.3), the linear Stern-Volmer plots of *q_FM_*/*ϕ_FM_* against *c* of gradient *K_CM_* (13) confirm the validity of the procedure, which corresponds to assuming

$$\phi_{FM}^{T}=\left(1-a\right)\phi_{FM}$$ (31)

This relation neglects the *secondary fluorescence* resulting from the self-absorption. Allowing for this, the author [11, 35] has shown that

$$\phi_{FM}^{T}=\frac{\left(1-a\right)\phi_{FM}}{1-a\phi_{FM}}$$ (32)

which approximates to (31) when *aϕ_FM_* ≪ 1, and that

$$\tau_{M}^{T}=\frac{\tau_{M}^{\prime }}{1-a\phi_{FM}}.$$ (33)

**Figure 3:**
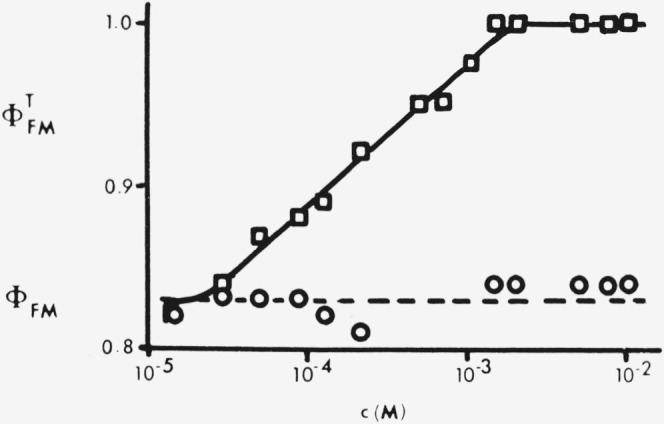
*9,10*-diphenylanthracene in benzene. Front-surface observation at *λ*_ex_=^:^365 nm. Technical fluorescence quantum yield $\phi_{FM}^{T}$ (+) and true fluorescence quantum yield *ϕ_FM_* (o) against molar concentration *c.* Data from Melhuish [36].

Relation (33) is considered to be generally valid. Relation (32) is considered to he valid for the transmission and 90° geometries. It is also valid for the reflection geometry, except for specimens of high *ϕ_Fm_*. Under the latter conditions the secondary fluorescence contributes markedly to the observed fluorescence intensity, so that $\phi_{FM}^{T}>\phi_{FM}$ in reflection, although $\phi_{FM}^{T}<\phi_{FM}$ in transmission as predicted by (32). Figure 3 plots Melhuish’s observations [36] of $\phi_{FM}^{T}$ as a function of c for 9,10-diphenylanthracene (DPA) in benzene solution, excited at 366 nm with front-face observation. Due to secondary fluorescence $\phi_{FM}^{T}$ increases from *q_FM_* = 0.83 in very dilute solution to $\phi_{FM}^{T}=1.0$ at *c* ⩾ 1.5×10^−3^*M*. Correction for self-absorption and secondary fluorescence, using a much more complex relation than (32), showed that *ϕ_FM_* ^=^ 0.83 ±0.02 over the whole range of *c*, thus demonstrating that DPA is immune to concentration quenching [36].

The secondary fluorescence contribution to $\phi_{FM}^{T}$ increases with decrease in the excitation penetration depth *d_ex_.* Berlman’s [12] choice of an intense absorption hand for excitation (*λ*_ex_ = 265 nm for DPA) minimizes *d*_ex_. This minimizes the effect of self-absorption on $F_{M}^{T}\left(\bar{v}\right)$, but it also maximizes the effect of secondary fluorescence on $\phi_{FM}^{T}$. To reduce the latter, a weak absorption region should be chosen for excitation, and *c* should be kept as low as possible.

To summarize, there are no particular problems in determining *ϕ_FM_* for (a) very dilute solutions (b) more concentrated solutions observed in the transmission or 90° geometries, and (c) more concentrated solutions of *ϕ_FM_*<~0.3 observed in the reflection geometry. The effects of self-absorption and secondary fluorescence are, however, difficult to compensate in concentrated solutions of high *ϕ_FM_* observed in the reflection geometry. One simple solution is to abandon the reflection geometry and to observe such systems in the more tractable transmission geometry. The alternative is to utilize one of the numerous mathematical relations, some simple [11, 35], some complex [27, 36], which have been developed to describe self-absorption and secondary fluorescence.

### 4.3. Fluorescence Standards

Melhuish [36] proposed the use of a 5 × 10^−3^*M* solution of quinine bisulphate (QS) in 1*N* sulphuric acid as a fluorescence standard. From careful measurements he obtained *ϕ*_FM_ = 0.510 for *c* = 5×10^−3^*M* increasing to *q_FM_* = 0.546 at infinite dilution at 25 °C. The value of *ϕ_FM_* at any other concentration can be evaluated using the Stern-Volmer relation (13). The QS solution is stable under prolonged irradiation, its fluorescence is not quenched by dissolved air (unlike most aromatic molecules), and it has a very small over-lap of the absorption and fluorescence spectra. It suffers from three minor disadvantages:

- concentration quenching;
- the temperature coefficient of *ϕ_FM_* is about − 0.25 percent per degree over the range 10° to 40° C; and
- sulphuric acid is not a conventional solvent for aromatic molecules and this necessitates using the refractive index correction in (25).

Nevertheless the QS standard, and various secondary standards derived therefrom, have been adopted in this and many other laboratories [28, 37]. Quinine is the fluorescent entity, and the use of quinine sulphate in place of the bisulphate does not appear to effect the values of *q_FM_* and *ϕ_FM_* [28]. Unfortunately many authors have chosen to use 0.1 *N* sulphuric acid as the solvent, rather than 1 *N* as recommended by Melhuish [36], while assuming his fluorescence quantum yield values to be unchanged. There is evidence that *ϕ_FM_* increases by 6–8 percent on increasing the solvent normality from 0.1 *N* to 1 *N* [28].

Table 2 lists comparative data on *τ_M_* and *q_FM_* for very dilute solutions of several aromatic compounds obtained using the QS standard [16–18]. The consistency of the data from three different laboratories is gratifying. The close agreement between the experiment values of *k_FM_*(= *q_FM_*/*τ_M_*) and the theoretical values of $k_{FM}^{t}$ from (23) for several compounds shows the error in q*_FM_* for the QS standard to be small. Gelernt et al. [36] have recently calorimetrically determined *q_FM_* for QS in 1 *N* sulphuric acid at 25 °C. The calorimetric value of *q_FM_* = 0.561 (±0.039) agrees satisfactorily with the fluorimetric value of *q_FM_* = 0.546 [34]. Other fluorescence standards have been discussed by Demas and Crosby [28].

Berlman [12] used a 10^−3^*M* solution of 9,10-diphenyl-anthracene (DPA) in cyclohexane, excited at 265 nm (an absorption maximum) and observed in reflection, as a fluorescence standard. Under these conditions the DPA solution has a technical fluorescence quantum yield of $\phi_{FM}^{T}=1.0$, due to self-absorption and secondary fluorescence, although the true fluorescence quantum yield is *ϕ_FM_* = *q_FM_* = 0.83 (±0.02) (fig. 3). Relation (25) requires that the specimen and standard be compared under identical conditions of excitation and optical density, so that the 10^−3^*M* DPA solution standard is only suitable for observations of $\phi_{FM}^{T}$ on concentrated solutions in reflection geometry. The QS standard is more versatile since it does not limit the specimen concentration or optical geometry.

Berlman [12] observed $\tau_{M}^{T}$ with heterochromatic excitation and $F_{M}^{T}\left(\bar{v}\right)$ with monochromatic excitation (these parameters need to be observed under identical conditions for (32) and (33) to be applicable [35]). He evaluated $\phi_{FM}^{T}$ by comparison with $F_{R}^{T}\left(\bar{v}\right)$ for the DPA standard observed under similar conditions, although the optical densities and excitation wavelengths of the specimen and standard appear to have differed. Apart from the usual hot band elimination and some 0–0 band attenuation, $F_{M}^{T}\left(\bar{v}\right)$ approximates to the molecular spectrum $F_{M}\left(\bar{v}\right)$. $\phi_{FM}^{T}$ and $\tau_{M}^{T}$ do not correspond to *q_FM_* and *τ_M_*, as implicitly assumed by Berlman [12], who used them to “evaluate” *k_FM_.* They require correction for self-absorption and secondary fluorescence to obtain *ϕ_FM_* and *τ_M_*, and these parameters need correction for concentration quenching to obtain *q_FM_* and *τ_M_*. Birks [1] tried to correct Berlman’s $\phi_{FM}^{T}$ data [12] by renormalizing them to *q_FR_* = 0.83 for DPA, but this procedure has since been shown to be invalid [23].

It is of interest to note the effect of substituting different fluorescence parameters in the relations used to evaluate *k_FM_* and *k_IM_.* From (3a), (13), (21), (22), (32) and (33)

$$\frac{q_{FM}}{\tau_{M}}=\frac{\phi_{FM}}{\tau_{M}^{\prime }}=k_{FM}$$ (34)

$$\frac{\phi_{FM}^{T}}{\tau_{M}^{T}}=\left(1-a\right)k_{FM}$$ (35)

$$\frac{\left(1-q_{FM}\right)}{\tau_{M}}=k_{IM}$$ (36)

$$\frac{\left(1-\phi_{FM}\right)}{\tau_{M}^{\prime }}=\frac{\left(1-\phi_{FM}^{T}\right)}{\tau_{M}^{T}}=k_{IM}+k_{CM}c.$$ (37)

An ideal fluorescence standard for aromatic solutions should

- have no self-absorption,
- have no concentration quenching,
- be in a common solvent suitable for other aromatic molecules (to eliminate the refractive index correction),
- be readily available as a high-purity material (or be insensitive to impurities), and
- be photochemically stable.

QS satisfies (iv) and (v) and it approximates closely to (i), but it does not satisfy (ii) and (iii). DPA meets criteria (ii)–(v), but it exhibits strong self-absorption. To minimize self-absorption in an aromatic hydrocarbon solution it is necessary that *S*_1_ is a ^1^*L_b_* state, so that the *S*_0_ → *S*_1_ absorption is weak, and not a ^1^*L_a_* state, giving strong *S*_0_ → *S*_1_ absorption, as in DPA [1]. There are two hydrocarbons which exhibit no concentration quenching (ii), have *S*=^1^*L_b_* so that self-absorption (i) is reduced, and satisfy (iii) and (v). These compounds, *phenanthrene* and *chrysene*, merit consideration as fluorescence standards. They can be obtained, but are not yet readily available, as high- purity materials (iv).

*Aromatic excimers* satisfy all the criteria for a fluorescence standard, since they have no self-absorption (i) or concentration quenching (ii) [1]. In concentrated solutions the excimer spectrum $F_{D}\left(\bar{v}\right)$ can be readily distinguished from the attenuated monomer spectrum $F_{M}^{T}\left(\bar{v}\right)$ [34], although the presence of the latter may be undesirable. It can be eliminated by the use of a pure liquid or crystal. A *pyrene crystal* has *ϕ_FD_* = *q_FD_* = 1.0 at low temperatures and *ϕ_FD_* = *q_FD_* = 0.65 at room temperature, a hroad structureless fluorescence spectrum between 400 and 550 nm with a maximum at 470 nm, and no self-absorption in any optical geometry [1]. It would appear to be an ideal crystal fluorescence standard.

## Figures and Tables

**Table 1:** Terminology of photo physical processes

Process	Organic	Inorganic
		
1. Luminescence,		
(a) spin-allowed	Fluorescence (*F*)	Fluorescence
(b) spin-forbidden	Phosphorescence (*P*)	Fluorescence
(c) thermally-activated delayed	E-type delayed fluorescence	Phosphorescence
2. Radiationless transition		
(a) spin-allowed	Internal conversion (*IC*)	
(b) spin-forbidden	Intersystem crossing (*ISC*)	
3. Vibrational relaxation	Vibrational relaxation (*VR*)	
4. Radiationless transition plus vibrational relaxation	*IC* (or *ISC*) and *VR*	Multiphonon process

**Table 2:** Fluorescence lifetimes (*τ_M_*) and quantum efficiencies (*q_FM_*) of very dilute solutions

Compound	Solvent	*τ_M_* (ns)	*q_FM_*	$k_{FM}/k_{FM}^{t}$	Ref.
					
Quinine Bisulphate	1*N* H_2_SO_4_	20.1	0.54	0.73	[17]
	1*N* H_2_SO_4_	19.4	.54	.75	[18]
Perylene	benzene	4.9	.89	.93	[17]
	benzene	4.79	.89	.90	[16]
	benzene	5.02	.89	.90	[18]
Acridone	ethanol	11.8	.83	1.02	[16]
	ethanol	12.5	.825	1.05	[18]
9-Aminoacridine	ethanol	13.87	.99	1.15	[16]
	ethanol	15.15	.99	1.02	[18]
9,10-Diphenyl anthracene	benzene	7.3	.85	0.99	[17]
	benzene	7.37	.84	.98	[18]

## Footnotes

1 Figures in brackets indicate the literature references at the end of this paper.
